# Production and Characterization of Flame Retardant Leather Waste Filled Thermoplastic Polyurethane

**DOI:** 10.1021/acsomega.3c09074

**Published:** 2024-02-14

**Authors:** Sumeyye Ustuntag, Nida Cakir, Aysegul Erdem, Ozkan Ozmen, Mehmet Dogan

**Affiliations:** † Department of Textile Engineering, Erciyes University, Kayseri 38039, Turkiye; ‡ Department of Fashion Design Trabzon Vocational School, Karadeniz Technical University, Trabzon 61080, Turkiye; § Turkish Standards Institute, Ankara 06100, Turkiye; ∥ Department of Industrial Design Engineering, Erciyes University, Kayseri 38039, Turkiye; ⊥ Erciyes Teknopark, Hematainer Biotechnology and Health Products Inc, Kayseri 38010, Turkiye

## Abstract

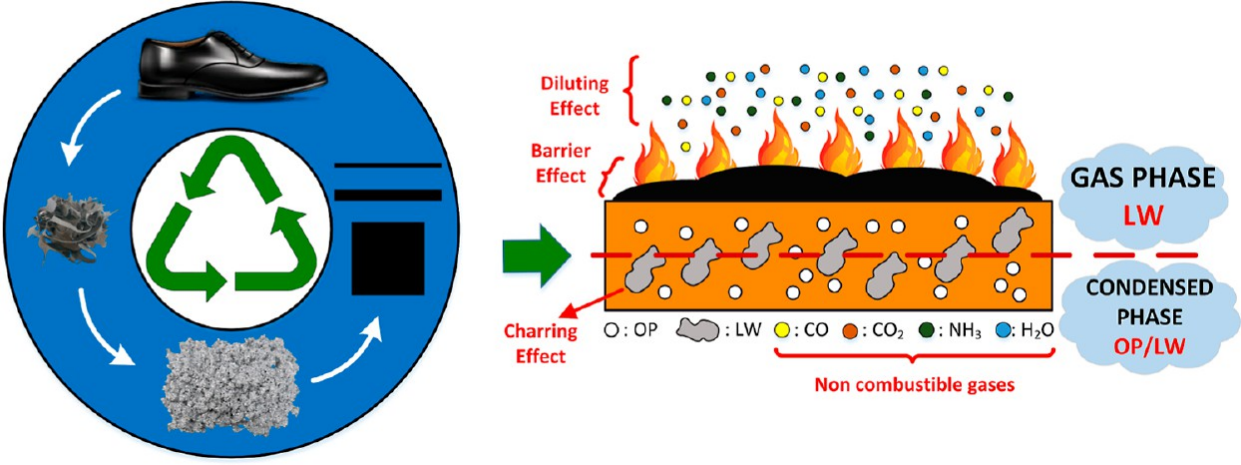

Discovering new applications for discarded materials, such as leather waste (LW), has proven to be an effective approach to an ecofriendly and sustainable production. The manufacture of halogen-free flame retardant LW containing thermoplastic polyurethane (TPU)-based samples containing an organic phosphinate (OP)-based flame retardant additive would represent an advance in this area. The effects of LW and OP levels on the thermal, flame retardant, and tensile properties of the samples using thermal gravimetric analysis (TGA), limiting oxygen index (LOI), vertical UL-94 (UL-94 V), mass loss calorimetry, and tensile tests have been assessed. OP is highly effective in LW-filled TPU. The highest UL-94 V rating of V0, LOI value of 31.4%, the lowest peak heat release rate (93 ± 3 kW/m^2^), and total heat evolved (49 ± 2 MJ/m^2^) values are obtained with the use of 20 wt % OP. OP is primarily promoted through the creation of a compact intumescent residue structure in the condensed phase. LW exhibits an adjuvant effect by producing nonflammable gases in the gas phase and raising the residual yield in the condensed phase. The most remarkable effect of the LW presence is observed in fire performance index (FPI) and fire growth rate (FIGRA) values. The highest FPI value of 0.49 sm^2^/kW and the lowest FIGRA value of 0.91 kW/m^2^s are observed with the use of 20 wt % LW.

## 1 Introduction

The leather industry, which is one of the most polluting and resource consuming sectors, produces a huge amount of solid waste in the different forms of hair, shavings, leather dust, and split offcuts. Various biological, chemical, thermal, and immobilization methods are mainly performed for the valorization of these solid wastes, which can be used in the production of absorbent materials, biodiesel, biogas, and biopolymers.^1−3^ The presence of solid wastes in the polymer composites greatly reduces the mobility and leaching of chromium. Accordingly, novel applications for tanned leather scraps in polymer-based composites are of great interest.^1,4^ The immobilization method has been used for the valorization of tanned split-off cuts in the shoe industry.

The inherent fibrous structure, high protein content, thermal insulating character, and sound deadening properties of leather waste (LW) increase its potential use in composite applications. LW is used as a biobased filler in various thermoplastic matrix materials including, poly(vinyl chloride),^5,6^ polyacrylonitrile,^7^ poly(vinyl alcohol),^8−10^ ethylene vinyl acetate,^11,12^ polyethylene,^13−15^ acrylonitrile butadiene rubber,^16^ polyamide,^17^ thermoplastic starch,^18^ poly(lactic acid),^19^ and thermoplastic polyurethane (TPU).^20−23^ The mechanical behaviors of the resulting filled materials have been a focus. In the limited number of studies, the effect of LW on the electromagnetic shielding and flame-retardant properties of the materials has been investigated. Enhancements in wear resistance,^1,6,11^ hardness,^5,6,11,23^ elastic modulus,^11,15,18,19^ tensile strength,^13−15,18−20^ flexural strength,^15^ tear strength,^23^ toughness,^21,22^ electromagnetic shielding,^7−9^ and flame retardancy^10,12^ have been observed.

TPU, a segmented block copolymer, has tunable final properties through the alteration of the kind and ratio of hard and soft segments. With a diversity of grades and special properties, it finds numerous applications in many engineering fields such as medical, automobile, construction, etc.^24,25^ However, its inherent flammability limits its wider application. Accordingly, the improvement of the flame-retardant performance of TPU has been of major interest.^26−28^ Phosphorus-based compounds are highly effective in both filled and unfilled TPU systems. Aluminum diethyl phosphinate (AlPi) and mixtures of cooperative agents are considered as highly effective nonhalogenated flame retardant additive in pure and filled TPU.^29−35^ A commercially available AlPi-based mixture has been examined as a flame retardant additive for LW-filled TPU.

TPU was selected as the matrix material because of its polar character and reasonable low processing temperature. The polar character of TPU facilitates good interfacial adhesion and dispersion of LW. Low processing temperature limits the oxidation of Cr (III) to Cr (IV) which is more detrimental to the environment.^1^ Accordingly, the resulting composites have a lower negative effect on the environment than do compositions generated under more stringent conditions. Owing to the high protein content of LW, it can be used as a biobased adjuvant filler in flame-retardant applications. High protein bearing fillers have been used for this purpose in TPU in conjunction with different flame-retardant additives.^1,36,37^ The potential use of LW as an adjuvant filler in the production of environmentally friendly flame retardant TPU composites has been explored. The levels of LW and flame-retardant additive on the thermal, flammability, and tensile properties of the samples using thermogravimetric analysis (TGA), limiting oxygen index (LOI), vertical UL 94 (UL-94 V), mass loss calorimetry (MLC), and tensile tests have been examined.

## 2 Experimental Studies

### 2.1 Materials

TPU, acquired as Desmopan 1045D from Marmara Polimer, Turkiye, has a density of 1.22 g/cm^3^ and a shore hardness D of 46. The synergistic mixture Exolit OP 1312, which is composed of AlPi, melamine polyphosphate (MPP), and zinc borate (ZnB) with an optimized char-forming ability, was kindly supplied from Clariant, Germany.^38^ The mixture has a density of 1.6 g/cm^3^ and a phosphorus content ranging from 18.7 to 19.7%. Cowhide leather straps containing <3 mg/kg of chromium VI were generously supplied by Mekap Deri ve Ayakkabı Sanayi Ticaret A.Ş.

### 2.2 Production of Samples

**Figure 1:**
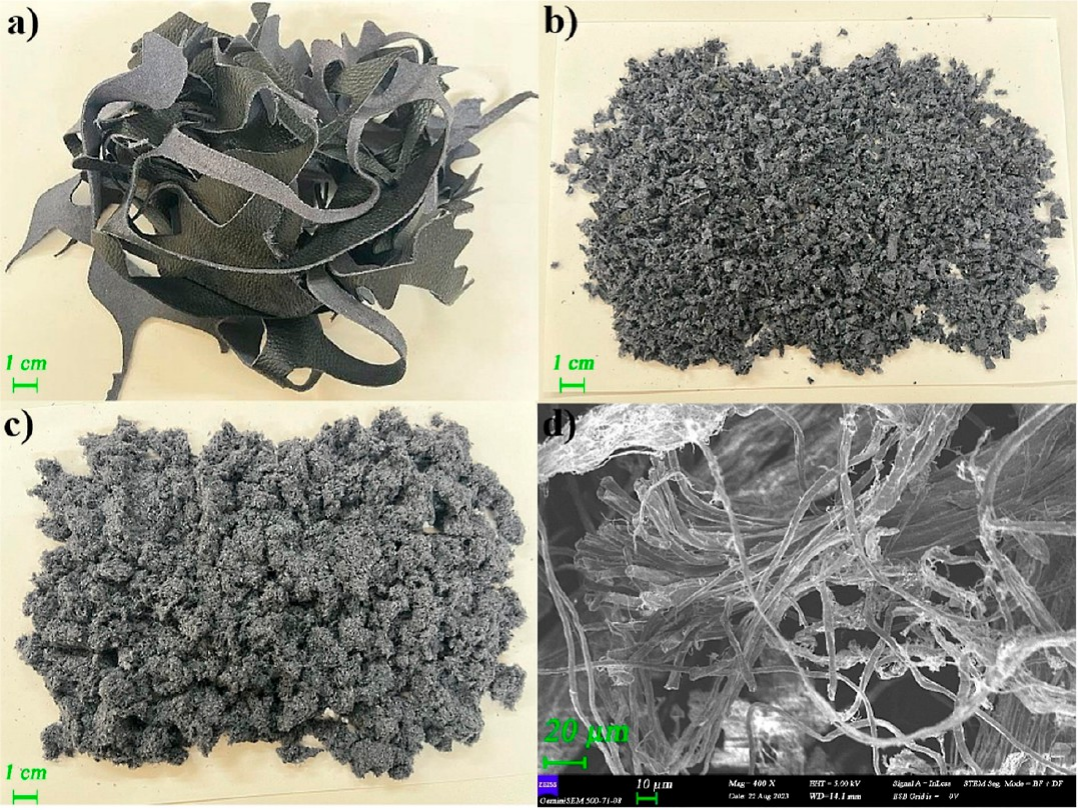
Photographs of (a) leather straps, leather particles after (b) first and (c) second grinding processes, and (d) SEM image of ready to use LW.

Leather straps underwent a two-step pulverizing process to reduce their size. Initially, a high-speed plastic crusher (SG-230F) was employed to grind leather straps. Subsequently, leather particles were further ground using a Fritsch Pulverisette 19, Germany. The photographs of leather straps (a), leather particles after first (b) and second (c) grinding processes, and SEM images of ready to use LW (d) are illustrated in Figure 1. Before the compounding process, TPU was also ground with the pulverizer used in the second step to ensure a more homogeneous mixture. Prior to the extrusion and molding processes, TPU, LW, OP, and extrudates were dried at 60 °C for 12 h to remove the physically absorbed water. The compounding process took place in a twin screw extruder (Gülnar Makina, Kayseri, Turkiye) operating at 100 rpm with an extruder temperature profile of 50–190–195–200–200–195 °C from hopper to die. Flammability (LOI, UL-94 V) and tensile test samples were molded using a laboratory scale injection-molding machine (DSM Xplore 12 mL Micro-Injection Molder, Netherlands). The molding process was conducted at a barrel temperature of 210 °C and a mold temperature of 30 °C. MLC test samples were produced using a laboratory-scale hot-press (GULNAR MAKINA, Istanbul, Turkiye) for 3 min at 175 °C. After the samples were produced, they were stored in desiccator until the characterization tests were performed. Accordingly, dry samples were characterized without any conditioning. The flame retardant performance of OP1312 was examined under the constant loading of LW (20 wt %). OP1312 was used in three different concentrations of 5, 10, and 20 wt %. Under constant loading of OP1312 (20 wt %), the effect of LW amount (5, 10, and 20 wt %) on the final properties of samples was also investigated. For sample coding, the abbreviations TPU, LW, and OP were used for thermoplastic polyurethane, leather waste, and Exolit OP1312, respectively. The code TPU/20LW/10 OP indicates the sample containing 20 wt % LW and 10 wt % Exolit OP1312.

### 2.3 Characterization Methods

TGA tests were conducted on individual components (TPU, LW, and OP1312) as well as their blends. The experiments were carried out using a Hitachi-High Tech STA-7300 instrument with a heating rate of 10 °C/min from room temperature to 800 °C under a N_2_ flow of 50 mL/min. The samples (weighing about 10 ± 0.5 mg) in granule form were inserted in an alumina pan. The uncertainty in TGA experiments for the decomposition temperatures and residue yields is ±3 °C and 5%, respectively. LOI tests were performed on the samples with the dimensions of 130 × 6.5 × 3.2 mm^3^ according to the standards of ASTM D2863. UL-94 V tests were performed on the samples with dimensions of 130 × 13 × 3.2 mm^3^ according to the ASTM D3801 standard. MLC test was performed on the samples with dimensions of 100 × 100 × 3 mm^3^ using Mass Loss Cone with thermopile attachment (Fire Testing Technology, U.K) under the heat flux of 35 kW/m^2^ according to ISO 13927 standard. The uncertainty in MLC experiments is below 5%. Tensile tests were carried out on a Shimadzu AG-X universal testing machine equipped with 50 kN load cell, in accordance with ASTM D 638 standard at room temperature with a crosshead speed of 50 mm/min. The tensile strength and percentage elongation at break values were recorded, and the outcomes were averaged over five samples, and standard deviations were calculated for accuracy. The tensile fracture surfaces of samples, the microstructures of LW and the residues remained after the MLC test were investigated with SEM (FEI Quanta 400F). The sample surfaces were covered with gold to obtain the conductivity.

## 3 Results and Discussion

### 3.1 Thermal Decomposition of Additives

**Figure 2:**
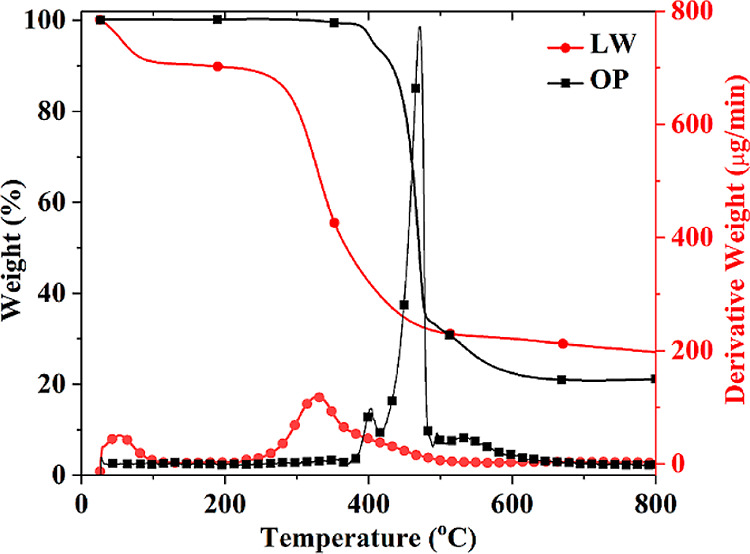
TGA and DTGA graphs of the additives.

The TGA technique has been widely utilized to evaluate the thermal properties of materials. Thermal decomposition characteristics of the additives are investigated under a nitrogen atmosphere, and the corresponding data are given in Table 1. Figure 2 displays the TGA and derivative TGA (DTGA) graphs. LW decomposes in two steps at 54 and 330 °C. The first step is attributed to the loss of physically absorbed water, while the second step, primarily driven by the decomposition of the collagen molecule, initiates around 250 °C and ended at 520 °C. The broad nature of the second peak stems from the uneven cross-linking structure of LW in the presence of Cr (III) along with the volatilization of low-molecular-weight compounds.^39,40^ LW contains mainly carbon (50–55%), nitrogen (15–20%), and oxygen (19–26%) and trace amounts of sulfur and chromium.^41,42^ It leaves mainly 26.9% carbonaceous residue at 800 °C. The main gaseous products of CO_2_, CO, ammonia, water, methane, ethane, and numerous minor products depending on the type of tanning process were formed during the decomposition of LW.^39,43−45^

**Table 1:** TGA Data of the Additives and Samples

sample	*T*_5%_ (°C)^a^	*T*_max1_ (°C)^b^	*T*_max2_ (°C)^b^	*T*_max3_ (°C)^b^	residue yield calc. (%)^c^	residue yield exp. (%)^d^
**LW**	60	54	330			26.9
**OP**	407	400	471	530		21.2
**TPU**	302	341	400			8.5
**TPU/20LW**	298	326	394		12.2	13.2
**TPU/20LW/5OP**	283	324	384		12.8	14.8
**TPU/20LW/10OP**	278	322	379		13.5	19
**TPU/20LW/20OP**	272	319	381		14.7	20.2
**TPU/5LW/20OP**	284	320	384		12.0	12.5
**TPU/10LW/20OP**	274	322	384		12.9	18.8

a Temperature at 5% weight loss.

b The maximum decomposition rate temperatures.

c Char yield at 800 °C (calculated).

d Char yield at 800 °C (experimental).

OP, consisting of AlPi, MPP, and ZnB, decomposes just starting from 375 °C and ending at 605 °C in multiple steps occurring at 400, 471, and 530 °C. The distinct decomposition steps of individual components within OP were not clearly observed, likely due to interactions among the components. It leaves 21.1% residue at 800 °C. The detailed mechanistic studies show that the intact AlPi molecule, diethylphosphinic acid, and melamine are mainly observed in the gas phase, and boron/aluminum phosphate based residue is mainly formed in the condensed phase.^46,47^

### 3.2 Thermal Decomposition of Samples

**Figure 3:**
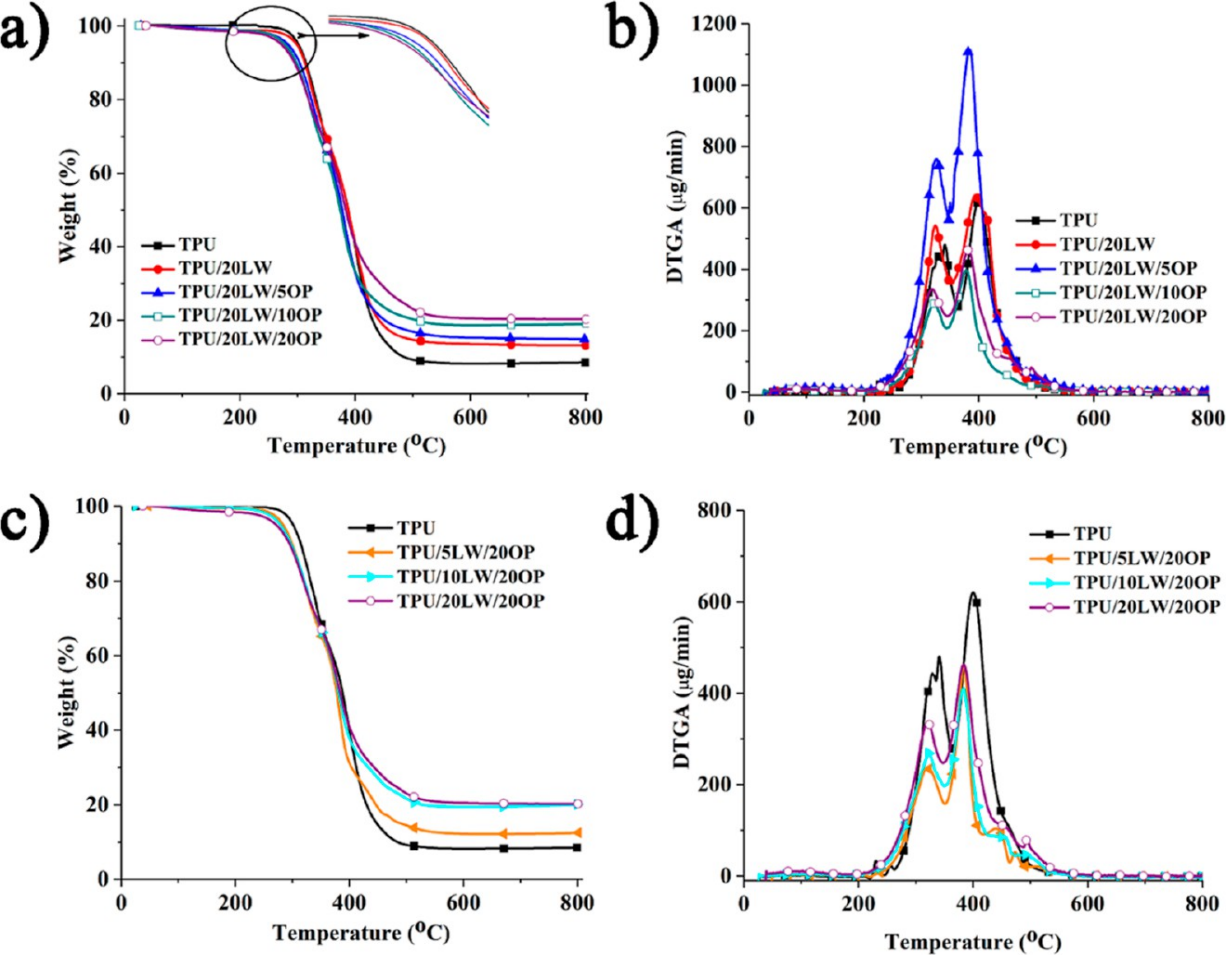
TGA and DTGA curves of the samples; (a) TGA graphs, (b) DTGA graphs with increasing OP amount, (c) TGA, and (d) DTGA graphs with increasing LW amount.

Thermal decomposition characteristics of the neat and filled TPU samples are investigated using TGA under an inert atmosphere. The corresponding TGA and DTGA curves are illustrated in Figure 3, and the relevant data are given in Table 1. TPU undergoes two step decomposition process, with the maximum weight loss rates at 341 and 400 °C. It leaves 8.5% carbonaceous residue at 800 °C. The initial step involves the decomposition of the urethane bond, while the second step arises from the decomposition of polyols in the soft segments through C–C and C–O bond cleavage.^26,48^ Despite LW exhibiting low initial thermal stability (*T*_5%_) at 60 °C, the *T*_5%_ of TPU shows a slight decrease when 20 wt % LW is added, attributed to the drying process eliminating physically absorbed water before the extrusion process. All studied samples exhibit a two-step decomposition pattern similar to pure TPU. In truth, the samples exhibit multiple decomposition steps that overlap instead of just having two steps. The maximum decomposition temperatures of both steps reduce with the inclusion of LW. The reduction in *T*_max1_ is more distinct owing to the low thermal stability of LW. With the addition of LW, the residue yield increases from 8.5 to 13.2% due to the high inherent char forming ability of LW.

Under the constant loading of LW (20 wt %), the *T*_5%_, and *T*_max1_ values consistently reduce with the increasing OP amount. The *T*_max2_ value reduces up to 10 wt % OP addition. Notably, the presence of OP accelerates the decomposition of both LW and TPU. Moreover, the addition of OP also enhances the residue yield as the added amount increases. A comparison between the calculated and experimental residue yields reveals that the experimental values significantly exceed the calculated ones. The interaction among OP, LW, and TPU is suggested. The observed trends may stem from the formation of acidic compounds of diethyl phosphonic acid (AlPi) and poly(phosphoric acid) (MPP) during the decomposition of OP. These acidic compounds play a role in expediting the hydrolysis of LW and TPU, promoting the carbonization reactions in the condensed phase. Similar findings, the reduction in thermal stability and the improvement in char amount, are also observed with the use of AlPi,^29,31−33^ MPP^49,50^ in TPU, and AlPi,^30^ MPP,^36^ and melamine phytate^37^ in a protein-based filler containing TPU.

Under the constant loading of OP (20 wt %), the *T*_5%_, the value reduces steadily with the increasing amount of LW. However, no prominent effect of LW is noted on *T*_max1_ and *T*_max2_ values in the presence of OP. The residue yield increases as the added amount of LW increases. An intriguing observation emerges when the calculated and experimental residue yields are compared. The experimental residue yield is very close to the calculated one in the 5 wt % LW containing sample. However, as the LW amount increases, the difference between calculated and experimental yields becomes more pronounced. The presence of OP is likely to favor the carbonization of LW more effectively than TPU due to the high heteroatom (N, O) content of LW.^51,52^

### 3.3 Mass Loss Calorimeter Studies

**Figure 4:**
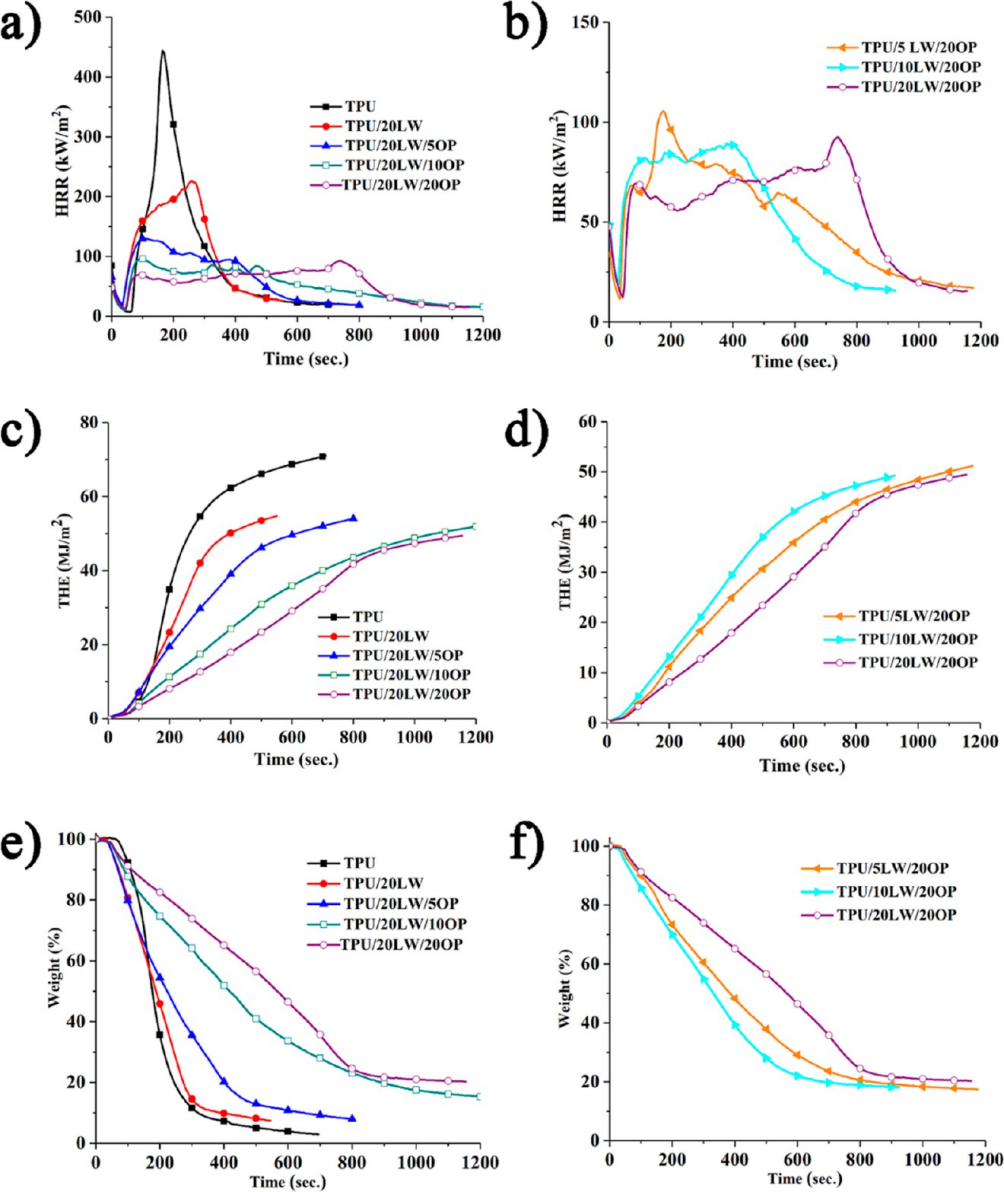
(a) HRR, (c) THE, and (e) weight vs time graphs of the samples with increasing OP amount; (b) HRR, (d) THE, and (f) weight vs time graphs of the samples with increasing LW amount.

**Figure 5:**
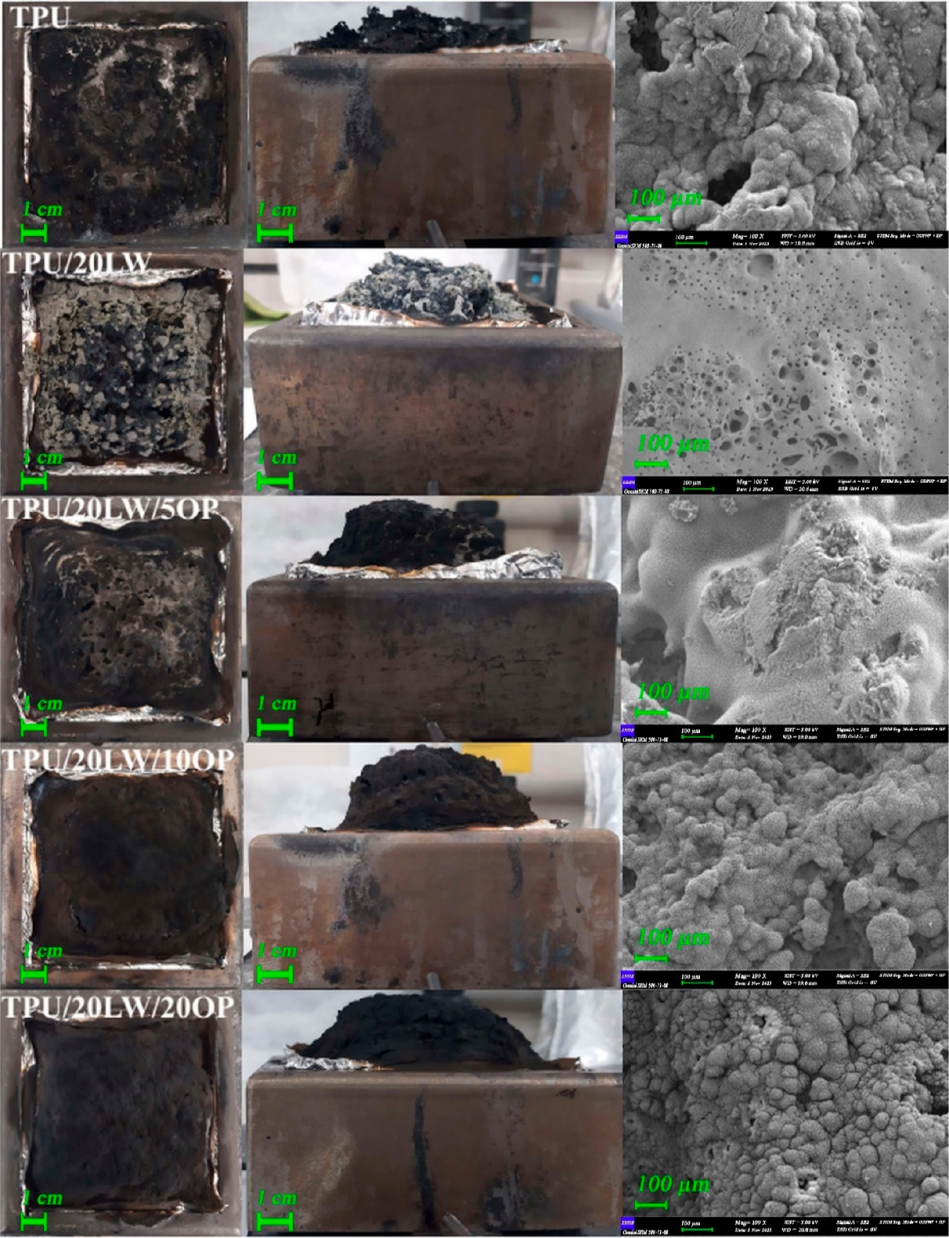
Photographs and SEM images (100×) of the residues.

**Figure 6:**
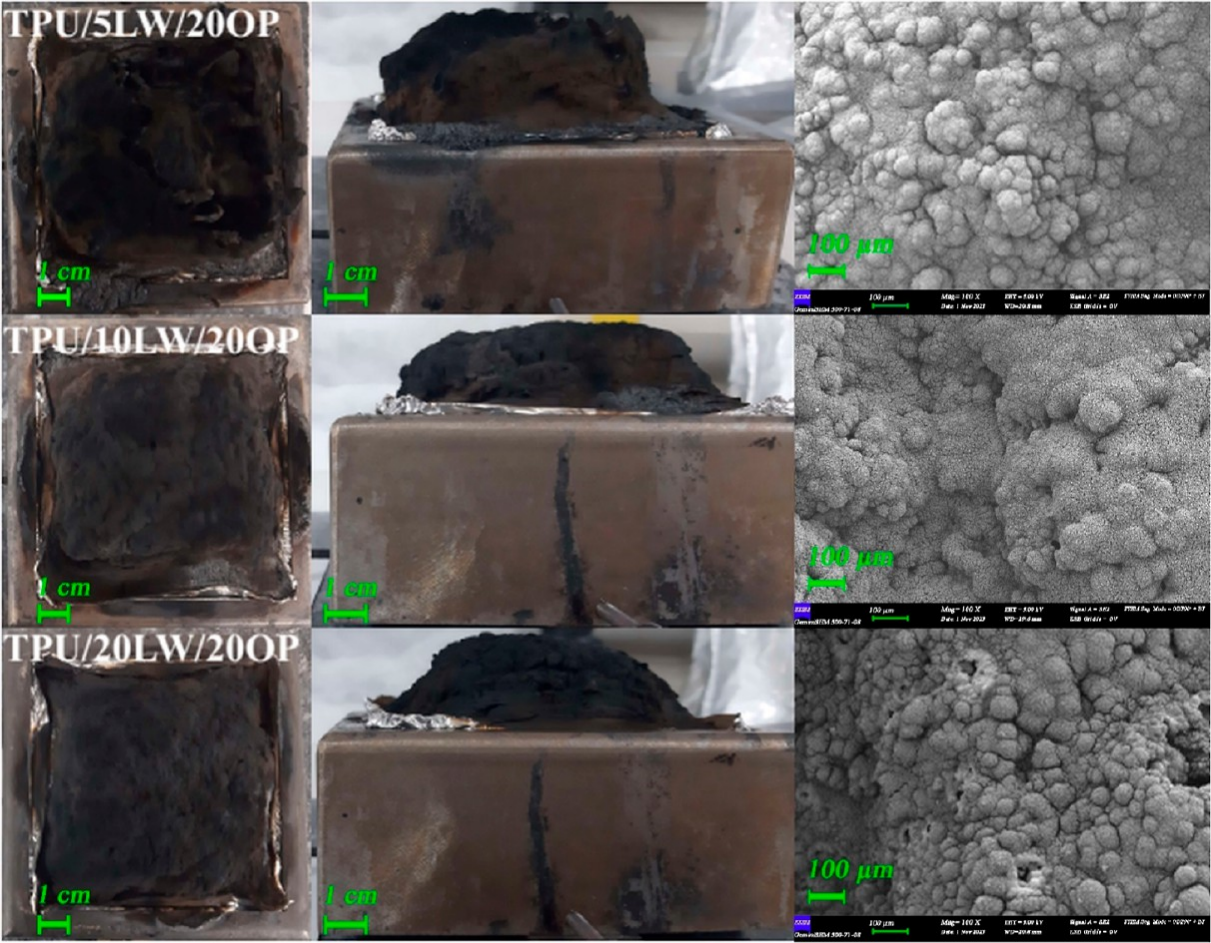
Photographs and SEM images (100×) of the residues.

The fire performances of polymer-based composites are frequently assessed and compared using valuable data such as time to ignition (TTI), peak heat release rate (pHRR), total heat evolved (THE), fire performance index (FPI), fire growth rate (FIGRA), average mass loss rate (AvMLR), average effective heat of combustion (AvEHC), residue yield, etc. achieved from mass loss calorimeter (MLC) tests. The related MLC data of the samples are given in Table 2. The HRR, THE, and weight versus time graphs of the samples are depicted in Figure 4. The photographs and SEM images (100×) of the residues are shown in Figures 5 and 6.

**Table 2:** MLC Data of the Samples

sample	TTI (sec)	pHRR (kW/m^2^)	THE (MJ/m^2^)	AvMLR (g/s)	AvEHC (MJ/kg)	FPI (sm^2^/kW)	FIGRA (kW/m^2^s)	residue (%)
**TPU**	64	444 ± 6	67 ± 2	0.09	14.0	0.14	2.68	2.9
**TPU/20LW**	33	226 ± 4	55 ± 2	0.12	10.6	0.15	1.74	7.5
**TPU/20LW/5OP**	34	129 ± 7	52 ± 2	0.06	12.5	0.26	1.51	8.0
**TPU/20LW/10OPp**	44	96 ± 3	51 ± 2	0.04	13.6	0.45	1.17	15.2
**TPU/20LW/20OP**	46	93 ± 3	49 ± 2	0.04	12.9	0.49	0.91	20.3
**TPU/5LW/20OP**	35	106 ± 7	51 ± 2	0.04	13.3	0.33	1.11	17.5
**TPU/10LW/20OP**	30	90 ± 3	50 ± 2	0.04	13.2	0.33	1.26	18.1

TPU has a TTI value of 64 s and leaves 2.9 wt % carbonaceous residue at the end of the test. As depicted in Figure 5, neat TPU forms an uneven and loose char structure, leading to rapid burning with a distinct, sharp HRR peak after ignition. The addition of LW shifts the TTI value earlier owing to the low thermal stability and the dark gray color of the LW (see Figure 1) enhancing radiant heat absorption. Consequently, the quantity of volatile flammable compounds needed for ignition is reached in earlier time. The decrease in TTI value is reported with the inclusion of protein-based fillers.^1,37,53^ Due to the reasons stated above, AvMLR value increases from 0.09 to 0.12 g/s. pHRR, THE, and AvEHC values reduce with the addition of 20 wt % LW. Figure 5 reveals the formation of more residue (7.5 wt %) with moderate intumescent character containing large holes with the addition of LW. pHRR value decreases at about 50% compared to neat TPU owing to the barrier effect of the formed char structure. However, 14% reduction in the THE value is observed due to the enhanced char formation. The decrease in AvEHC value indicates the gas phase action of LW via fuel dilution owing to the formation of nonflammable gases CO_2_, CO, ammonia, and water.^39,43−45^ FPI and FIGRA are considered comprehensive fire safety evaluation parameters. FPI is determined by dividing TTI to pHRR, while FIGRA is calculated as the maximum value of HRR/tHRR.^54,55^ The higher FPI and lower FIGRA indicate higher product safety rank. The addition of LW has a negligible effect on the FPI value, whereas the FIGRA value reduces sharply.

The addition of OP leads to an increase in TTI value, particularly noticeable at concentrations of 10 and 20 wt %. Figure 5 illustrates that samples containing OP form a highly intumescent residue structure. The char structure of the 5 wt % OP-containing sample exhibits numerous holes, which diminish as the OP concentration increases, resulting in a more compact residue. All OP containing samples exhibit HRR curve of thick char forming materials depicting an initial rise in HRR until the formation of an efficient char, followed by a consistent decrease as the thickness of residual layer increases.^56^ As the OP concentration increases, both the pHRR and THE values reduce. pHRR value reduces by about 43, 58, and 59% with the addition of 5, 10, and 20 wt % OP in comparison to the TPU/20LW sample, respectively. THE value steadily decreases at about 5, 7, and 11% with increasing OP amount. These reductions primarily stem from a decrease in fuel source with enhanced residue formation and the barrier effect of compact residue structure with a notably intumescent character. The addition of OP sharply reduces the AvMLR value from 0.12 to 0.04 g/s up to 10 wt % addition due to the formation of the compact structure. However, the further addition (20 wt %) does not change the AvMLR value. FPI value steadily increases with increasing OP content and the highest value of 0.49 sm^2^/kW is achieved with the addition of 20 wt % OP. The addition of OP causes steady decrease in FIGRA with increasing amount. These results collectively highlight the significant impact of OP on the fire behavior and safety characteristics of the TPU composite.

Under constant loading of 20 wt % OP, THE, and AvEHC values steadily decrease and the residue yield increases with increasing amount of LW. The reduction in THE values arises from the improved residue yield. The reduction in AvEHC stems from the flame dilution effect of nonflammable decomposition products of LW as stated above. The prominent effect of LW is observed on the FPI and FIGRA values. The addition of LW improves the fire safety rank of the samples. The lowest highest FPI and the lowest FIGRA values are observed in 20 wt % LW samples.

### 3.4 Flammability Properties

**Figure 7:**
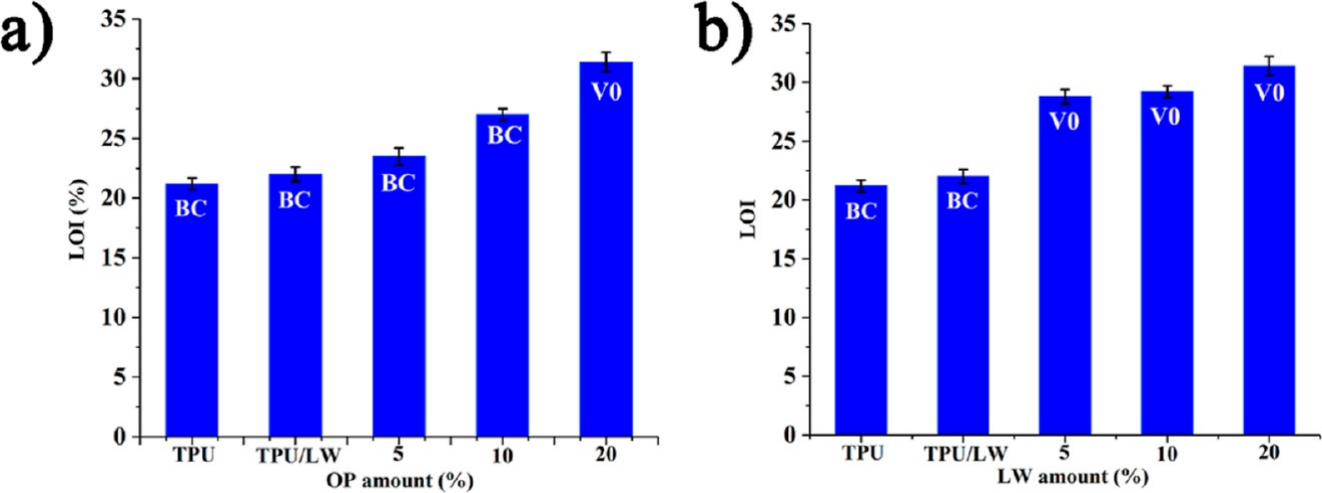
LOI and UL-94 ratings of the samples (a) with increasing OP amount and (b) with increasing LW amount.

The flammability characteristics of the samples are evaluated with LOI, and UL-94 V tests. The related LOI results with standard deviations and UL-94 V results are given in Figure 7. TPU burns to clamp in the UL-94 V test and has a 21.2% LOI value. The addition of 20 wt % LW does not change the UL-94 V rating, and a negligible increase in LOI value (22%) is observed. With the addition of OP, the LOI value steadily increases, reaching the highest LOI value of 31.4% with the addition of 20 wt % OP. Twenty wt % OP is required to get the highest UL-94 V rating of V0. Comprehensive experimental studies suggest that OP is a highly effective flame retardant additive, primarily acting in the condensed phase through the formation of a compact residue with a highly intumescent character. Under constant loading of OP (20 wt %), all samples get V0 rating and LOI value steadily increases from 28.8 to 31.4%. This slight enhancement in LOI value is proposed to be attributed to the enhanced residue formation and fuel dilution via the formation of nonflammable gases of carbon monoxide, carbon dioxide, ammonia, and water.

### 3.5 Tensile Properties

**Figure 8:**
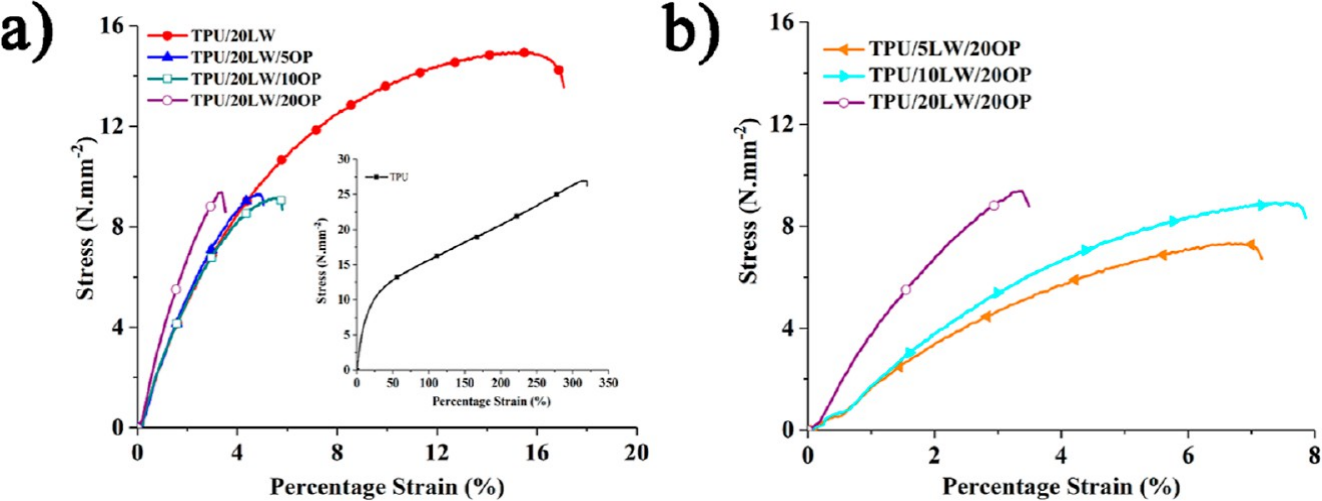
Stress–strain curves of the samples (a) with increasing OP amount and (b) with increasing LW amount.

**Figure 9:**
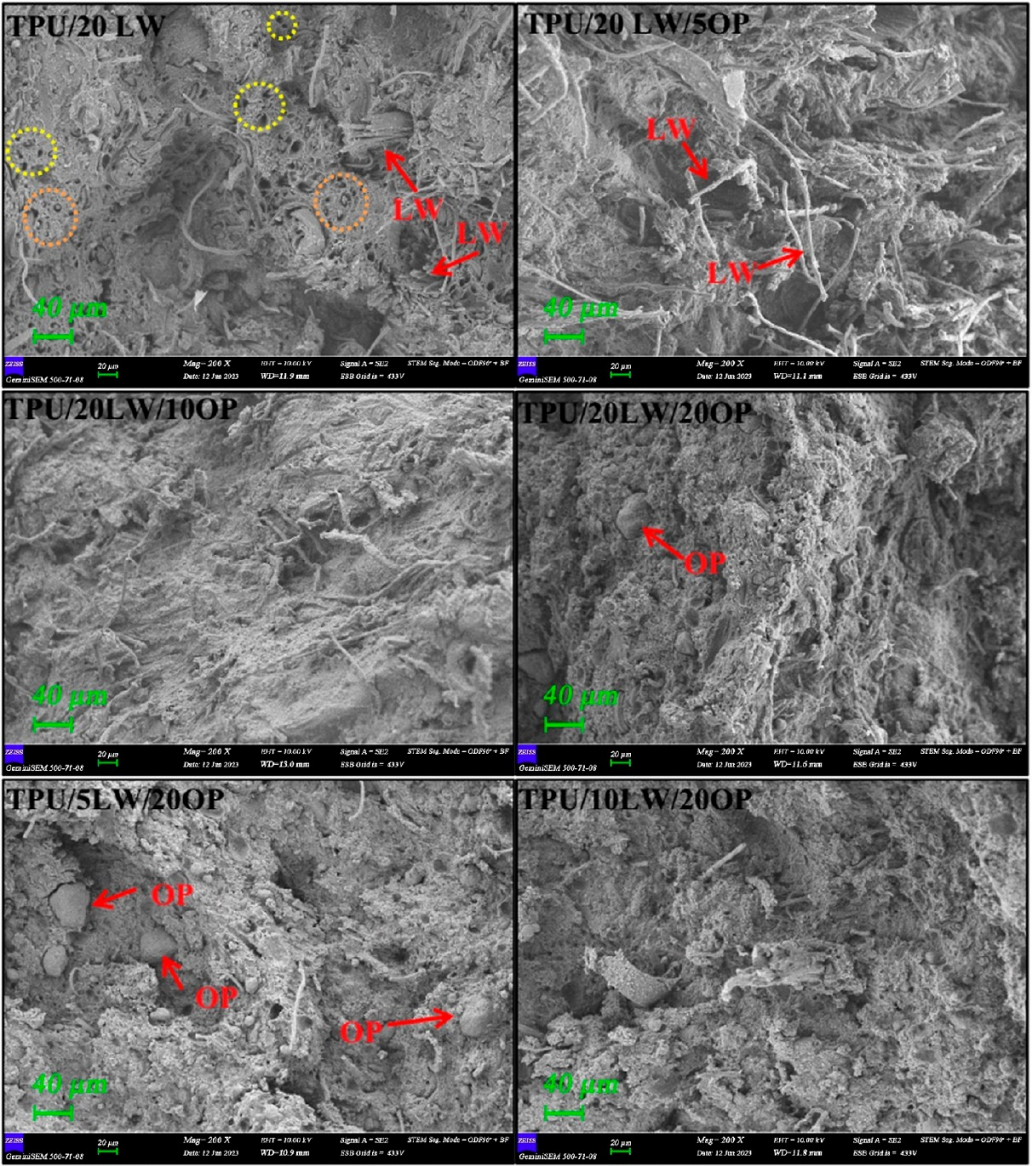
Tensile fracture surfaces of samples.

The representable stress–strain curves of neat TPU and samples are shown in Figure 8 and the corresponding data are listed in Table 3. SEM analyses are conducted on the tensile fractured surfaces of samples. The relevant SEM images at 200× magnification are shown in Figure 9. The representable embedded fibers, debonded fibers, LW, and OP particles are highlighted in orange dashed circle, yellow dashed circle, and red arrows, respectively. According to Figure 8, neat TPU fails in a ductile manner with large elongation. With the addition of 20 wt % LW, the tensile strength and percentage strain at break values reduces at about 45 and 95%, respectively. Figure 9 reveals that LW particles are uniformly dispersed in TPU with a good interface adhesion. The large and coarse LW particles act as stress concentration centers and initiate crack. Consequently, load bearing capacity of the samples reduces, leading to premature failure. With the addition of OP, tensile strength reduces approximately 40% with respect to the TPU/20LW sample. However, negligible changes in tensile properties are observed with increasing OP concentration. Under constant loading of OP, a slight steady increase in tensile strength is observed with an increasing amount of LW, attributed to the fibrous structure of LW (see Figures 1 and 9). These findings collectively suggest that the incorporation of LW and OP has notable effects on the mechanical properties of the TPU composites. The large and coarse LW particles contribute to stress concentration and crack initiation, while the fibrous structure of LW may provide some improvement in tensile strength when combined with OP.

**Table 3:** Tensile Properties of the Samples

sample	stress (N/mm^2^)	strain (%)
**TPU**	26.9 ± 3.1	320 ± 18
**TPU/20LW**	14.9 ± 2.2	17 ± 1.3
**TPU/20LW/5OP**	8.9 ± 0.7	5.2 ± 0.4
**TPU/20LW/10OP**	8.7 ± 0.7	6 ± 0.6
**TPU/20LW/20OP**	8.6 ± 0.6	3.6 ± 0.8
**TPU/5LW/20OP**	6.7 ± 0.6	7.2 ± 0.6
**TPU/10LW/20OP**	8.3 ± 0.6	7.9 ± 0.6

## 4 Conclusions

This study deals with the adjuvant effect of LW in OP containing TPU. The parameters of LW and OP amount are examined on the thermal, flame retardant, and tensile properties of TPU-based samples. According to TGA results, OP enhances the char formation of both TPU and LW. The residue forming ability of OP is higher for LW rather than TPU. According to fire retardant test results, OP is highly effective in LW-containing TPU samples. Twenty wt % OP is needed to get highest UL-94 rating of V0 and LOI value of 31.4%. OP predominantly shows its flame-retardant action in the condensed phase through the formation of a compact intumescent char structure. LW acts as an adjuvant biobased filler by increasing residue yield in the condensed phase and by fuel dilution via forming nonflammable gases in the gas phase. The lowest pHRR, THE, FIGRA and the highest FPI values are obtained in 20 wt % LW- and OP-containing sample. The results collectively indicate that the addition of LW and OP contributes to enhanced flame retardancy and improved fire safety properties of the TPU composite. The incorporation of LW and OP has a notable effect on the mechanical properties of the TPU composites. The large and coarse LW particles contribute to stress concentration and crack initiation, while the fibrous structure of LW may provide some improvement in tensile strength when combined with OP. The examined specimens exhibit potential applications in situations requiring high flame retardancy performance alongside minimum mechanical properties.
